# Awe and anxiety for cancer cells: connecting scientists and patients in a holistic approach of metastasis research

**DOI:** 10.1186/s40900-023-00498-3

**Published:** 2023-09-26

**Authors:** Hildert Bronkhorst, Wytske M. van Weerden, Eline M. Bunnik, Hub Zwart

**Affiliations:** 1 Erasmus School of Philosophy, Erasmus University Rotterdam (EUR), Rotterdam, The Netherlands; 2 Department of Experimental Oncology, Erasmus Medical Centre (Erasmus MC), Rotterdam, The Netherlands; 3 Department of Medical Ethics, Philosophy and History of Medicine, Erasmus Medical Centre (Erasmus MC), Rotterdam, The Netherlands

**Keywords:** Cancer metastasis, Patient and public involvement, Scientific and existential life world, Societal workshop, Holistic

## Abstract

**Background:**

Metastatic cancer is often experienced by patients as a death sentence. At the same time, translational scientists approach metastasis also as an interesting phenomenon that they try to understand and prevent. These two sides of the same coin do not mask the considerable gap that exists between the laboratory world of scientists and the life world of patients. Funding agencies nowadays increasingly demand researchers to be responsive to the values and priorities of patients and public. One approach to bridge this gap and to increase the impact of science is patient and public involvement (PPI). A concise literature review of PPI research and practice in this paper revealed that although PPI is often deployed in translational health care research, its methodology is not settled, it is not sufficiently emancipatory, and its implementation in basic and translational science is lagging behind. Here, we illustrate the practical implementation of PPI in basic and translational science, namely in the context of HOUDINI, a multidisciplinary network with the ultimate goal to improve the management of metastatic disease.

**Methods:**

This paper reports on a societal workshop that was organized to launch the holistic PPI approach of HOUDINI. During this workshop, societal partners, patients, and physicians discussed societal issues regarding cancer metastasis, and contributed to prioritization of research objectives for HOUDINI. In a later stage, the workshop results were discussed with scientists from the network to critically review its research strategy and objectives.

**Results:**

Workshop participants chose the development of metastasis prediction tools, effective therapies which preserve good quality of life, and non-invasive tissue sampling methods as most important research objectives for HOUDINI. Importantly, during the discussions, mutual understanding about issues like economic feasibility of novel therapies, patient anxiety for metastases, and clear communication between stakeholders was further increased.

**Conclusions:**

In conclusion, the PPI workshop delivered valuable early-stage input and connections for HOUDINI, and may serve as example for similar basic and translational research projects.

**Supplementary Information:**

The online version contains supplementary material available at 10.1186/s40900-023-00498-3.

## Abstract (plain language summary)

Metastatic cancer is an aggravated form of cancer, that patients are afraid of. At the same time, cancer researchers are fascinated by this disease. Therefore, there is an apparent gap between how patients and researchers feel about cancer. If researchers wish to be most helpful to cancer patients, it is important to consult the patients and ask what they need and find important. This is also stimulated by agencies that financially support research projects. A possible way to do this is Patient and Public Involvement (PPI), in which not only scientists, but also patients and lay people are asked to provide input. It appears, however, that PPI is scarcely applied in basic science. In this article, we describe how a collaborative network of basic and translational cancer researchers, HOUDINI, intends to include the patients’ voice throughout the research progress and actively asked for input from patients, societal partners and physicians at the start of their project. These people discussed what themes they found most important to be researched by HOUDINI. Later, the HOUDINI researchers reflected on this. This example shows how PPI can be applied and how HOUDINI received valuable input for their research goals.

**Supplementary Information:**

The online version contains supplementary material available at 10.1186/s40900-023-00498-3.

## Background

In 1971, US President Richard Nixon launched his famous ‘War on Cancer’ [1]. Compared to the high expectations, however, it became clear that the complexities of cancer and the challenges facing cancer research had been underestimated. Although during recent years for many forms of cancer considerable progress has been achieved, notably in the treatment of the primary cancer, secondary cancers (metastases, where healthy tissues become affected by migrating tumour cells) are much more difficult to treat requiring extensive systemic treatment. As the mortality burden of cancer has shifted from the primary to the secondary tumour, the detection of metastasis is often experienced by patients as a death sentence. Our understanding of the mechanisms of metastasis suffers from significant gaps, such as why certain tissues are affected while others are not, and how to treat the cancer without harming the healthy organs. Also, the development and severity of the secondary disease are hard to predict, while it will strongly affect quality of life. Unfortunately, translational medical research (i.e. the process of taking a laboratory idea to clinical implementation that benefits patients), is a laborious and sinuous pathway that can last up to twenty years and has a success rate lower than one percent [2].

Besides scientific considerations, national science agendas, institutional research politics and societal priorities have been identified as factors that contribute to the winding road of research that is needed for clinical utility of new findings [3, 4]. Even though such preclinical translational research is curiosity-driven and aimed at understanding the biological mechanisms of disease, it has the complementary aim to offer beneficial solutions and to create impact. To further direct and speed up the translational process, research funding organisations increasingly demand researchers to incorporate the priorities, benefits, values, and concerns of societal stakeholders. This strategy is expected to result in research that is more responsive to the priorities and concerns of patients and more impact-orientated. But how to do this in practice? How to bridge the gap between the laboratory and the patient life world, between scientific and experiential knowledge, between scientific discourse and biomedical practice?

Both researchers and patients pursue the same goal, namely curing or alleviating cancer. Researchers might be drawn to basic cancer science by personal experiences with cancer or because they aim to make a contribution to understanding the mechanism of a disease that affects millions of people [5]. At the same time, many scientists are also driven by curiosity or by puzzle-solving motivations [6]. They take on an objective stand to the disease, which is reflected in the scientific biomedical literature: it provides no space for personal reflections but exclusively focusses on factual data. Researchers spend years or even decades on studying particular tumour cells. A lay person who visits a cancer laboratory may be struck by the care with which valuable tumour cultures are treated, the beautiful pictures of tumour cells that are taken, the knowledge concerning tumour cells that is developed based on daily interaction and even familiarity with them. For laboratory researchers, a growing, flourishing tumour cell may be something very positive. It may indicate that the experimental work is on the right path. Their dedication and care for the cancer cell is essential to understand it, but this experience seems the opposite of the existential lifeworld experiences of patients, especially patients who are informed that metastasis has been detected. Instead of curiosity, such a disconcerting message will give rise to anguish, especially given the uncertainties of metastatic disease and the difficulty to make reliable predictions [7]. In this paper we will argue that this apparent gap between the laboratory view on cancer and the lifeworld experience of cancer is bridgeable when both parties are willing to learn from one another, and experiences of patients and their caretakers are involved in the design of new research trajectories. As both researchers and patients pursue the same goal, the eradication of cancer, a better understanding of the existential impact of metastasis may help to align research into cell cultures with priorities of patients, for instance concerning early detection or the prevention of side-effects. The experiences of both researchers and patients can contribute to a more holistic and long-term view on cancer that bridges the gap between both apparently incompatible worlds of experience.

It has been argued that researchers, clinicians, patients and societal partners should participate in research to bring curiosity and impact closer together, preferably throughout the entire process [8, 9]. To make research that is directed towards understanding basic mechanisms of cancer more impactful, patients, physicians, and other stakeholders should not only be involved in the translation and implementation of the research, but from the very onset of research. Rather than seeing patients and physicians as end-users (recipients of insights, knowledge, therapeutic options, and other outcomes), the project objective should be to build an inclusive knowledge coalition, which incorporates the knowledge and experience of patients and caretakers, fostering co-construction and co-ownership.

This argument has been adopted as point of departure by a recently established research network of multidisciplinary researchers, named HOUDINI, a Holistic approach to UnderstanD and target the metastatic Niche. The HOUDINI network aims to connect scientists, physicians, business representatives, and patients in their mutual desire to tackle metastatic cancer research. The HOUDINI network focusses on breast and prostate cancer and employs an inclusive, holistic research approach. Reflecting on our experience as active participants in this network, we first describe HOUDINI’s efforts to foster interaction between scientists from various disciplines and between societal partners from diverse backgrounds in order to deepen our understanding of cancer metastasis by combining laboratory knowledge with lifeworld experiences. Secondly, we introduce patient and public involvement (PPI) as a relevant method to realise the research objectives of HOUDINI, and we will elucidate the PPI content with the help of two examples. Thirdly, as a case study, we describe and critically reflect on a societal workshop that was organized to obtain patient and public input before the launch of the HOUDINI research network. We conclude by summarizing our findings and recommendations for bridging the gap between scientific expertise and patient experiences in translational medicine. The content of this article is summarized in the GRIPP2 form (Additional file 1).

### HOUDINI: origin and objectives

The new cancer research network, HOUDINI, adopts an impact-oriented approach to translational medicine as its research strategy. The research network explores and identifies targets for the treatment of metastatic processes. While metastasis accounts for up to 90% of cancer-related deaths [10], most research has been directed at the tumour of origin, not at its metastases. Thus, our understanding of metastasis is limited compared to our insights in primary tumours. HOUDINI investigates how the organ tissue that is affected by metastasis changes in order to help understand how tumour cells survive and grow in the hostile environment of a healthy organ. Current treatments of metastatic diseases are often targeted at either tumour cells, blood vessels or the immune system. Instead, HOUDINI aims to define new therapy targets that interfere with all essential factors that support the metastatic niche*.* To this end, HOUDINI aims to follow a holistic biomedical approach by developing novel therapeutic modalities that at the same time are responsive to patient priorities and concerns. Implementation of such new treatment strategies into clinical management requires involvement of multiple stake holders, such as biomedical and technology experts, philosophers, bioethicists, health economists and health policy scientists that all participate in the network to strengthen the feasibility of the project and its societal relevance. On top of this, HOUDINI also actively involves patients, physicians, policy makers, pharmaceutical industry representatives, journalists, and the general public throughout the entire project to foster epistemic inclusion. The knowledge, values, and priorities brought up by these stakeholders are considered relevant to (re)define research questions creating impactful project results based on realistic expectations. By adopting this inclusive approach, involving both the industry and patients, the new metastasis therapies of the network are more likely to reach the market and be implemented in clinical practice, while they at the same time align with concerns and expectations of patients.

The holistic approach of HOUDINI was developed in response to the Convergence Strategy of the knowledge institutes involved—the Erasmus Medical Centre (Erasmus MC), the Technical University Delft (TU Delft) and The Erasmus University Rotterdam (EUR). The objective of the Convergence Strategy is to foster convergence and integration of biomedical, technical, and societal perspectives in research, while the metropolitan region of Rotterdam offers a living laboratory for interactive and inclusive research ("Convergence EUR, Erasmus MC, TU Delft | Erasmus University Rotterdam" [11]). TU Delft and EUR are both universities of international prominence, and Erasmus MC is one of the largest academic medical centres in Europa. These three knowledge institutes are geographically located in close proximity, and complementary in the sense that their respective foci are on science and technology (TU Delft), biomedical research (Erasmus MC) and social sciences and humanities (EUR). Whereas the collaboration between TU Delft and Erasmus MC will advance technological innovation in biomedical research, linking up with EUR is expected to allow consortia such as HOUDINI to make the societal dimension of the research (e.g., ethical, governance, and economic aspects) an integral part of their research approach. In that way, the Convergence Strategy aims to contribute to open and responsible science according to Anticipatory, Inclusive, Reflexive and Responsive (AIRR) research concept to address societal needs, values and concerns [12, 13].

### Benefits and challenges of PPI

To foster convergence, inclusion and societal participation in practice, HOUDINI adopted a PPI approach (Savory 2010), as an integral dimension of the work. PPI has become more and more common in health care research [14]. It allows lay citizens, patients as well as non-patients, to influence and shape health care research. PPI generates high-impact science, provided that the design of such involvement is based upon a carefully considered and valid methodology [9, 15]. Often, however, patients and public are either involved in the first stages of a research program, such as exploration, consultation, and prioritization of research goals, or in the final dissemination stage, which limits the relevance of their participation [16–18]. A more holistic approach to PPI, allowing it to become part of the full empirical cycle, seems therefore highly desirable as the next step.

The relevance and significance of PPI are advocated by two different kinds of arguments: deontological and consequentialist. Deontological arguments represent values such as democracy and emancipation: research should not be conducted *to, about,* or *for* patients, being autonomous subjects, but rather *with* or even *by* them. Consequentialist arguments assert that PPI advances the efficiency and impact of health research [19, 20]. PPI will benefit successful implementation of treatments through acceptance, commitment, and willingness to adhere to new therapies.

The geographical distribution of PPI research is very uneven across Europe: most publications originate from western Europe [21] where the values and means for PPI research are prevalent. The United Kingdom is a frontrunner in participatory research [20], mainly due to stimulation by funding bodies [22]. There is a wealth of initiatives in the field of patient and public participation, reflecting a movement from science-centred to application-centred research. The general process of PPI has been analysed in (meta) reviews, focussing on the overarching principles and strategies [23], patient partnership dimensions [24, 25], the relation to translational research [9], or the impact of PPI [20]. Other reviews discussed aspects of PPI, like its methodology [18, 26], its role during different stages of healthcare innovation [17], and its implementation across Europe [21]. Still other reviews concentrated on the role of PPI in a particular disease, such as cancer prevention [27], cancer treatment [15], or melanoma [28]. All in all, these papers cover a wide field of PPI activities, and present the current state of affairs regarding PPI research in medicine.

Despite growing consensus on the benefit and necessity of PPI research, the latest reviews still reveal a great variety in its principles, methods, results, and recommendations. First, several publications criticizes certain aspects of PPI or even the entire concept itself. The criticisms of PPI as discussed in the literature can be classified into three different categories. First, researchers who want to implement PPI run into several conceptual and practical issues. There is some ambiguity when it comes to conceptually defining PPI research. This is reflected for instance by the underdefined use of terms such as *involvement* and *participation*, since in practice the level of involvement or participation may differ, and it is important to define more precisely how stakeholders are genuinely *included i*n research rather than merely being *consulted*. Researchers who wish to use PPI in a conscientious manner, run into practical obstacles, such as time constrains, limited funding, and recruitment problems. In many cases, PPI activities can only be organized after the funding of a research proposal is secured. This sequence severely limits the influence that the public can exert on the course of the proposed research. Importantly, basic scientists are generally not educated in social sciences and humanities, which is needed to successfully implement PPI. There are also communication barriers between academics and clinicians on the one hand, and the public and patients on the other hand. For a fruitful PPI, both parties need to move out of their comfort zone, adapting a different language than they are used to [24, 29].

Second, a common concern about PPI studies is flawed or misdirected methodology [18]. Ad hoc design of PPI strategies results in poor data quality of limited reproducibility. Due to the current policy incentive to foster public participation some PPI activities are poorly planned and processed, and could become tokenistic box-ticking exercises [30]. The ensuing emphasis on measuring the tangible impact of PPI activities (focussed on quantitative short-term metric) may obfuscate an approach that is more focussed on the quality of long-term relationships and long-term objectives, which require a qualitative change of the research culture. If PPI is improperly performed, it results in silencing the voice of the public by confirming technocratic power structures, rather than being democratizing and emancipatory [31].

Third, although PPI has become more common in clinical research, its implementation in basic research is still limited due to proper education, time constraints, communication issues, and lack of guidance [32]. There is, however, also a more fundamental issue at stake. Since basic science is considered curiosity-driven, it is less obvious why patients and the public should be involved and it is even more challenging to determine how this could be optimally done, given for instance the unpredictable and esoteric nature of basic research [33]. Thus, for basic science network like HOUDINI, the challenge is to meaningfully involve patients and public on various levels, while avoiding the two pitfalls mentioned above.

These three categories that challenge PPI application have in common the lack of consensus, time and knowledge, which may be considered as characteristic for a field still in its infancy. The more effort is invested in educating PPI concepts, the more progress will be made through mutual learning. The Houdini project as our case study aims to contribute to this, notably by showing that participatory approaches such as PPI are not only relevant for applied research, but should also be included in basic, curiosity-driven research. There already exists a broad movement, supported both by initiatives from within the basic research field (bottom-up) and by policies and strategies implemented by research funding and research performing organisations (top-down), towards open and responsible science, open access, and democratization [34]. For instance, in the context of life sciences research, scientists are prompted to address the ethical, legal and social aspects (ELSA) or of their research [35]. During the past decade, in the context of European funding, much attention has been given, both conceptually and practically, to the concept of responsible research and innovation (RRI). RRI has been explicated in four dimensions: anticipation, inclusiveness, reflectiveness and responsiveness (the aforementioned AIRR concept) [12, 36]. The European commission currently prefers to focus on measurable outcomes in terms of the five RRI pillars: Ethics, Science Education, Gender Equality, Open Access, Governance and Public Engagement [37]. Researchers are still urged to move in the direction of PPI, for instance by funding agencies such as the Dutch NWO and the European Commission, where public involvement is not only a requirement for funding, but support and advice is offered as well [38]. Moreover, partly in response to these ‘top down’ initiatives by funding agencies, multiple approaches have been developed by the research field themselves to make participatory research happen, for instance in terms of methodology development or case studies research, building on experiences in previous and ongoing projects. Recent examples of such projects are found in synthetic cell research [39], fundamental biology [40] and pluripotent stem cell research [41]. In conclusion, the latest PPI conceptualization and policy agendas create awareness among basic scientists that PPI also within the basic scientific research field is becoming imperative, but still difficult to implement because clear guidelines and helpful precedents are lacking.

### PPI in cancer research

Having concisely reviewed the general situation in PPI research, we now take a closer look at two examples of PPI in practice that closely match the planned approach of HOUDINI, namely in the work of the ReIMAGINE Consortium and the James Lind Alliance priority setting partnerships.

The ReIMAGINE Consortium, aiming to develop new methods for prostate cancer screening and diagnosing, implemented a varied patient and public involvement and engagement strategy in their research program [42]. The voice of the patient and public was already included during the writing of the grant application. After the grant was awarded and the research program was funded, a PPI coordinator was appointed to lead a series of discussion groups with different patient and public participants to allow for a diverse input regarding design, data collection, analysis and dissemination of findings. The PPI coordinator was supported by a PPI sub-committee with established relationships between patient (organizations) of diverse background, which ensured regular and appropriate communication from science to public and vice versa. The committee went at great lengths to include minorities as well, among others by installing a specialist prostate cancer research group for black, Asian and minority ethnic communities that provided a platform for under-represented patients to come in contact with researchers. It used Twitter as an “invaluable platform for our research engagement”, as it lead to participation in online PPI activities and research engagement events a broader public [42]. The researchers conclude that a well-designed involvement structure is an absolute prerequisite to accomplish successful PPI. Sufficient (monetary) means are required to appoint a funded PPI coordinator and a set up a consistently meeting PPI committee. PPI should start already during the grant application process and should not finish before the research project has been completed.

The James Lind Alliance (JLA) is a British nonprofit organization established in 2004, that aspires to bring patients, caregivers, and clinicians together in priority setting partnerships (PSPs). JLA provides a methodological framework for these partnerships to identify and prioritize ‘evidence uncertainties’ for various diseases. Evidence uncertainties are defined by JLA as open questions in science for which “no up-to-date, reliable systematic reviews of research evidence addressing the uncertainty exist”, or “up-to-date systematic reviews of research evidence show that uncertainty exist” [43]. After an initial survey to collect input from patients, caregivers, and clinicians on open questions with respect to a specific disease, the input is categorized and collated. An interim survey is then employed to make a relevant preselection. Finally, a live workshop is organized with all stakeholders during which the uncertainties are prioritized in a top 10. In the follow-up, these data are disseminated in an academic journal and on the JLA website. Since 2004, top 10s of priorities have been formulated for a plethora of diseases, including prostate cancer (2010) and metastatic breast cancer (2018). The top 3 research priorities for prostate cancer were [44]:

1. How can overtreatment for prostate cancer be prevented by identifying and excluding the treatment of harmless tumours?
2. Is there a genetic marker for prostate cancer that would be both more sensitive and more specific than PSA serum level?
3. What can be done to delay or prevent the onset of hormone-independent prostate cancer?

For metastatic breast cancer the top 3 priorities were the following [45]:

1. What biomarkers or intrinsic features of the tumour can be used to identify response to specific treatments and dosing schedules?
2. What is the role of immunotherapy for metastatic breast cancer?
3. How can treatment resistance be delayed, and minimized?

Research uncertainty prioritization is a helpful tool to direct basic and translational medicine into the relevant and urgent direction. However, for long-term research programs such as HOUDINI, PPI should not be limited to the first phase of research, but be an integrative part of the full empirical circle. Such a structural societal alliance would be an enrichment to the existing priority setting partnerships, since a substantial number of decisions have to be made in later stages of research.

## Case study: a societal workshop for proposal enhancement

### Methods

Learning from these two initiatives, HOUDINI has been set to include PPI from the onset. To prepare the ground, an interactive workshop was organized for HOUDINI network members and its societal partners. The sixteen participants of the societal workshop were four members of the network (specialized in prostate cancer research, medical ethics, philosophy of science and health technology assessment), two prostate cancer patients, two representatives of Dutch cancer (patient) organizations, three urologists, one quality-of-life expert, three representatives from industry, and one health care journalist. A semi-structured interview was conducted afterwards to include the breast cancer perspective in this study as well. The workshop was not hosted in a hospital or university setting, hoping that participants would experience the ambience as a level playing field. The participants signed an informed consent form to ask permission for audio-recording the plenary discussion and publication of anonymized results.

After a general introduction of HOUDINI and its objectives, first a plenary discussion was organized around four themes: (1) first thoughts on cancer metastasis, (2) the balance between patient, science, ethics, and economy, (3) the desirability of screening for metastases, and (4) tissue donation by patients and healthy persons for scientific research. The participant’s input was collected by an interactive presentation followed by reflection on the results with the entire group. The participants were invited to respond to each other and learn from each other’s perspectives. After a break, breakout groups were organized to discuss these issues from a specific perspective: (1) patient, (2) business, (3) clinician, and (4) society. The consortium members joined the different breakout groups such that every group had three or four participants. These breakout groups were asked to formulate questions and issues that HOUDINI’s research should address. In a final plenary setting, the input from these groups was reviewed, and subsequently, targets for research were prioritized on the basis of a voting session.

## Results

### Societal issues in metastasis research

**Fig. 1:**
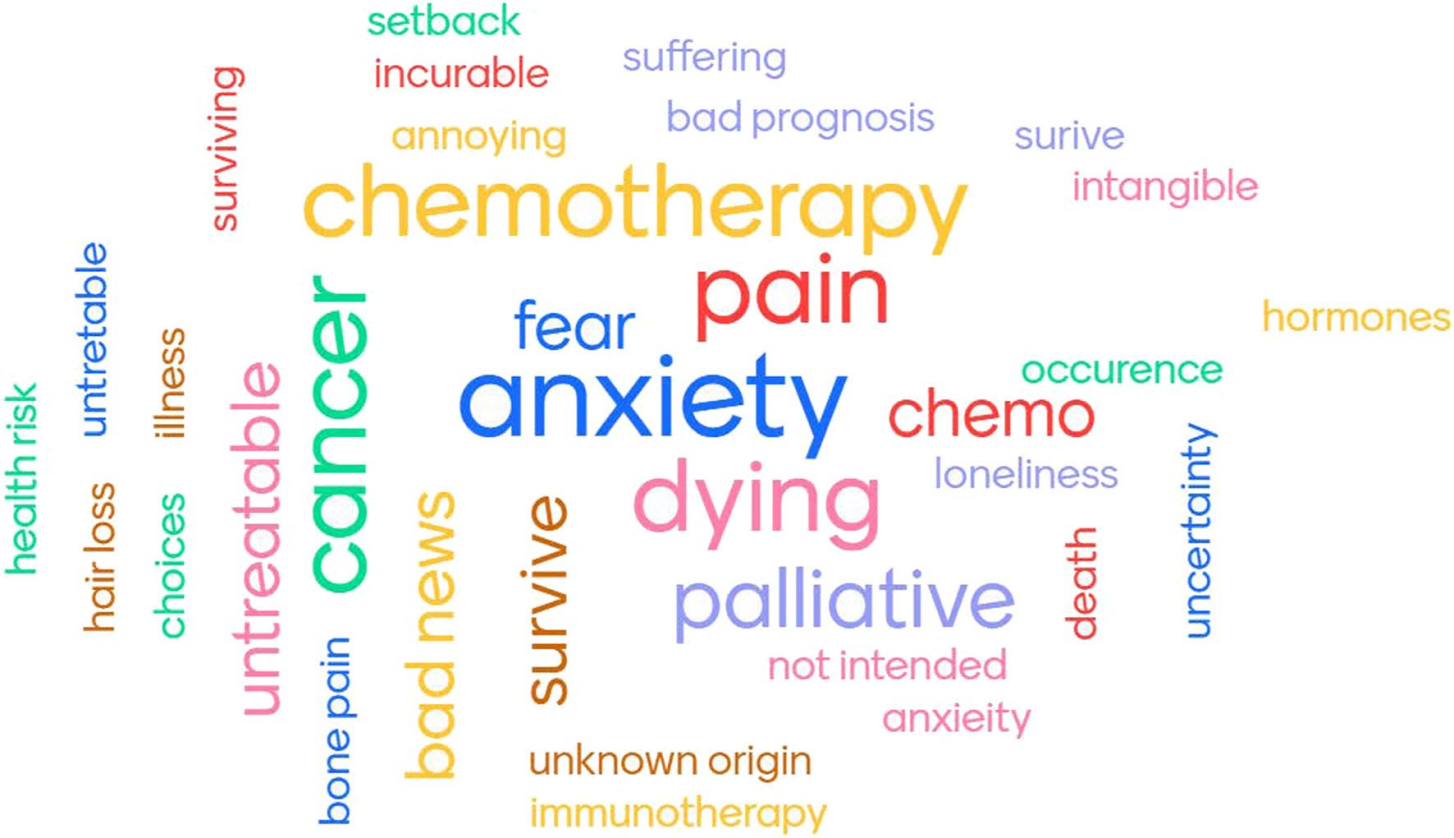
First thoughts on cancer metastasis from the workshop participants

The participants were first asked to fill in 1–5 terms that came to their mind first when thinking about metastases. Figure 1 shows the resulting word cloud. The font size is proportional to the number of times the word was entered. Central in the diagram are mostly existential concepts like ‘anxiety’, ‘pain’, ‘dying’, ‘survival’, and ‘untreatable’. The participants explained that according to the current status of cancer treatment, getting the diagnosis of a metastasized cancer evokes feelings of uncertainty and loneliness because there is often no curative therapy available. It is the general perception that metastatic disease is incurable, which indeed is correct, and thus untreatable and deadly, which in fact is a misconception that causes major distress. Also, the potential pain and expected reduction in quality of life which metastatic treatments entail is feared by the patients. Moreover, a primary tumour is confined to one place in the body, while a metastasized cancer is spread across the body and can be ‘everywhere’, which further adds to the uncertainty associated with metastasis. For patients, uncertainty seems the main reason for concern. As one physician phrased it: “Patients can deal with a bad diagnosis relatively well when their prognosis can be reliably defined, e.g. when they know that they have either three months or three years more to live. But if their prognosis is uncertain, e.g. somewhere between three months and three years, the uncertainty becomes unbearable”. Hence, an important objective of metastasis research should be the development of reliable prediction tools. The challenge for researchers is that most cancer cells never develop into a malignant tumour. Also, for many cancer types, it is currently not possible to predict if and when metastatic disease will develop in an individual patient. In the context of HOUIDINIs program, the discussion revealed that developing bioassays that can predict metastatic potential of a patient’s cancer is highly valued, especially if this can be connected to treatment response prediction.

**Fig. 2:**
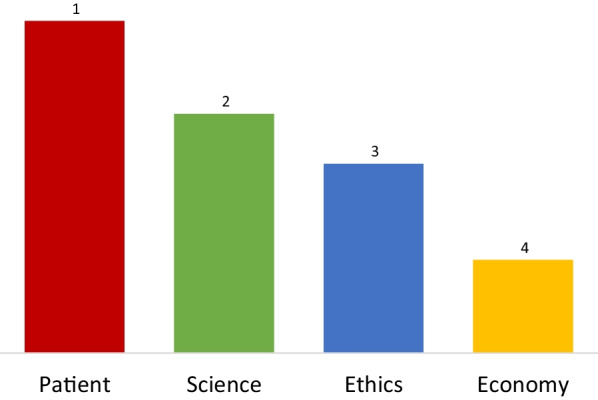
Prioritization of patient, science, ethics, and economy by the participants

A second topic that was addressed was the priority balance of patient, science, ethics, and economy. In a tetralemma poll (Fig. 2) the patient was put first by the participants, followed by science, ethics, and economy. A lively discussion of these results ensued. All participants agreed that by voting with their ‘heart’ the patient would be number 1. However, by voting with their mind, they also needed to take in account science, ethics, and the economy. It was agreed that the patient is the end, while science, ethics and economy are only means to serve the patient. The HOUDINI research plan fits in the current trend towards personalized therapies, which are tailored to the biological characteristics of individual patients and, therefore, are hoped to offer a better treatment with less side effects. However, this development comes with increasing health care costs, which imposes a heavy burden on the sustainability of health care organizations. The clinicians in the group indicated that they actually do not discuss the costs of therapies with their patients, because they do not wish the patient to realize that there is an economic aspect to his treatment. On the other hand, one of the patients indicated that there is already awareness of the economic burden that patients impose on the health care system, and that they would like to be informed about the financial impact of their treatments. All agreed that it is hard to balance between the individual patient and the entire health care system. The participants put forward several possibilities to reduce health care costs, which are important to the HOUDINI research plan. A metastatic risk prediction tool for individual patients would be very helpful. Also, it was emphasized by one of the participants that for some young patients or patients early after diagnosis three more months to live are a great gift, while for other (elderly) patients that already went through a long treatment trajectory, another three more months might not add much value. In some cases, patients may decide that it is more beneficial to spend their last days without having to suffer from side effects of aggressive, but still poorly effective medication. Finally, the participants addressed the high costs of effective drugs that isa direct consequence of the significant failures in drug development, which is supposed to be at least partly caused by cancer models used for compound selection that poorly reflect actual patients. In conclusion, HOUDINI should aim to develop more realistic metastasis models to improve the development of new therapies, which will also benefit and serve more efficient drug development.

The third theme of the discussion was the role of knowledge between patients, physicians, and the public in scientific research. A majority of the workshop participants agreed with the statement ‘I want to participate in research to prevent metastasis, even if I am not at risk’ (8.3 on the scale of 1–10, Table 1). The underlying question here is, whether it is desirable to always know a potential situation in as much detail as possible. Does knowledge have intrinsic value or is it only valuable when it has become relevant in terms of treatment options? In Western society, we are inclined to measure everything we can, possibly because it provides a feeling of control. But sometimes, more ‘knowledge’ will rather lead to more uncertainty or more concerns. For example, the best prostate specific antigen tests available have a very high sensitivity to measure the presence of micrometastases of prostate cancer. However, these metastatic cells cannot be located by scans, cannot be treated, do not cause illness, and are not yet known to pose any risk to the patient’s life. Should these very accurate test results be shared with prostate cancer patients when these cells do not yet have to be treated, if at all? If the results are shared, patients need to live with these concerns. If not, the patients have years of rest. As the measured prostate specific antigen in these very early stages of the disease may have little or no clinical significance, ‘knowing’ it does not make a difference to long-term prognosis. Nevertheless, in the workshop, patient participants indicated that they prefer to be informed on all results, as they could help the patient to take individual action to gain control by considering preventive measures, such as life-style changes. With respect to the HOUDINI research project, this discussion clarified that early prediction or detection tools to define (micro) metastasis are valued even if they cannot predict outcome. Such outcomes will offer patients a way to re-gain control to help beating the disease. Preferably, such diagnostic improvements should be accompanied by improved therapeutic possibilities or preventive (lifestyle) changes.

**Table 1:** Statements and mean voting results

Statement	Disagree–agree (1–10)
1. I want to participate in research to metastasis prevention research, even if I am not at risk	8.3
2. As patient I am open to tissue donation for scientific purposes	8.9
3. As healthy person I am open to tissue donation for scientific purposes	7.9

Finally, the workshop participants shared their thoughts on tissue donation for the purpose of scientific research. The patients would readily give their permission to take biopsies of organs in the context of a planned (medically necessary) surgery (8.9 on a scale of 1–10; Table 1). The patients underlined that they have an interest in scientific research that could result in new treatment options for their situation and for future patients. The participants voted 7.9 on a scale of 1–10 that they are also willing to donate tissue (blood, skin, small biopsies) as non-patients. The discussion that followed clarified that the willingness to donate tissue highly depends on the risk that comes with the procedure to sample the material. Low-risk blood sampling or biopsies are more likely to attract volunteers. The new organ-on-a-chip technology based on human tissues that the HOUDINI network will develop, should therefore also aim to develop minimal invasive biopsy procedures that are acceptable for patients as well as healthy volunteers.

### Societal questions for HOUDINI

To review the HOUDINI research plan from different angles, the workshop participants were divided in four groups focusing on either the patient, the societal, the clinical, or the business perspective. These groups came up with the following issues that HOUDINI should address.

1. The patient breakout group stressed the importance of clinical translation. Cancer metastasis research should be made relevant to patient populations as soon as possible. Knowledge itself is not the goal, but rather the application of this knowledge to improve the lives of patients. A reliable test to predict the risk of metastasis at a time of diagnosis, and a drug to help prevent metastases would be very helpful to them, especially if this treatment results in a higher quality of life. Quality of life is essential for many patients, so the adverse impact due to side effects of every intervention needs to be balanced against the benefits. Furthermore, basic research that contributes to a deeper understanding of metastasis is valuable, because it eventually allows for better-targeted treatments. HOUDINI’s research could result in a similar development, but now focused on metastases. Lastly, HOUDINI should focus on identifying effective lifestyle interventions for metastasis prevention because a change of lifestyle is a relatively feasible strategy for patients and may foster their sense of empowerment and agency.
2. According to the societal perspective breakout group, the HOUDINI research plan was not holistic enough yet. The participants of the workshop were mainly white and well-educated. In future meetings, it would be wise to reach out for a more inclusive representation of society. It is also important to embed HOUDINI’s research and potential therapies in the existing health care organization. There should be a clear pathway towards therapeutic application to ensure that the results of preclinical research can be used in the future. Moreover, it might be helpful if HOUDINI’s research enables us to better discern between patient subgroups, such that only patients that have increased risk of metastasis need to be monitored. Finally, communication is key in all aspects. For a holistic approach, a universal language needs to be developed that can be understood by patients, societal partners, business, clinicians, et cetera. Thus, HOUDINI should put much effort in enhancing communication to convey its relevance for society.
3. The clinician perspective breakout group asked for a better substantiation of the probability of HOUDINI’s hypotheses. Which processes will be studied? Will the metastases themselves be the main topic of interest, or the interaction between metastases and receiving tissues? Which diseases are to be targeted? With the focus on prostate and breast cancer, HOUDINI has a rather narrow scope. Moreover, for these cancer types, there is already a number of effective therapies available. Patients suffering from more aggressive tumors such as pancreatic cancer, are much more likely to benefit from new therapies against metastasis. Moreover, pancreatic cancer metastasis may be less demanding to study because their malignant progressing is much faster. A final important issue is the possible need for a tissue biobank. HOUDINI should realize that setting up a biobank for donated tissue is no easy task.
4. The business breakout group discussed the difficulty of collaboration between basic scientists and entrepreneurs. Basic science is a fundamentally open process, while business is more often interested in a cost-effective and profitable product. There are, however, investment funds or companies that aim to strengthen long-term sustainable economic growth, such as the Dutch *Nationaal Groeifonds*. It is essential that, in an early stage, HOUDINI consults and engages companies about the direction, purpose and final application of its research. Together, they should start market research on a possible application, considering the health care structure, feasibility, and desirability for the clinic. HOUDINI might also look for implementation of a broad user panel to achieve early platform standardization in terms of hardware software, sensors or imaging. The collaboration could be a dynamic process by adding or replacing expertise as needed. In short, the HOUDINI network should elaborate to its business partners what it aims for and how it will reach these goals.

**Fig. 3:**
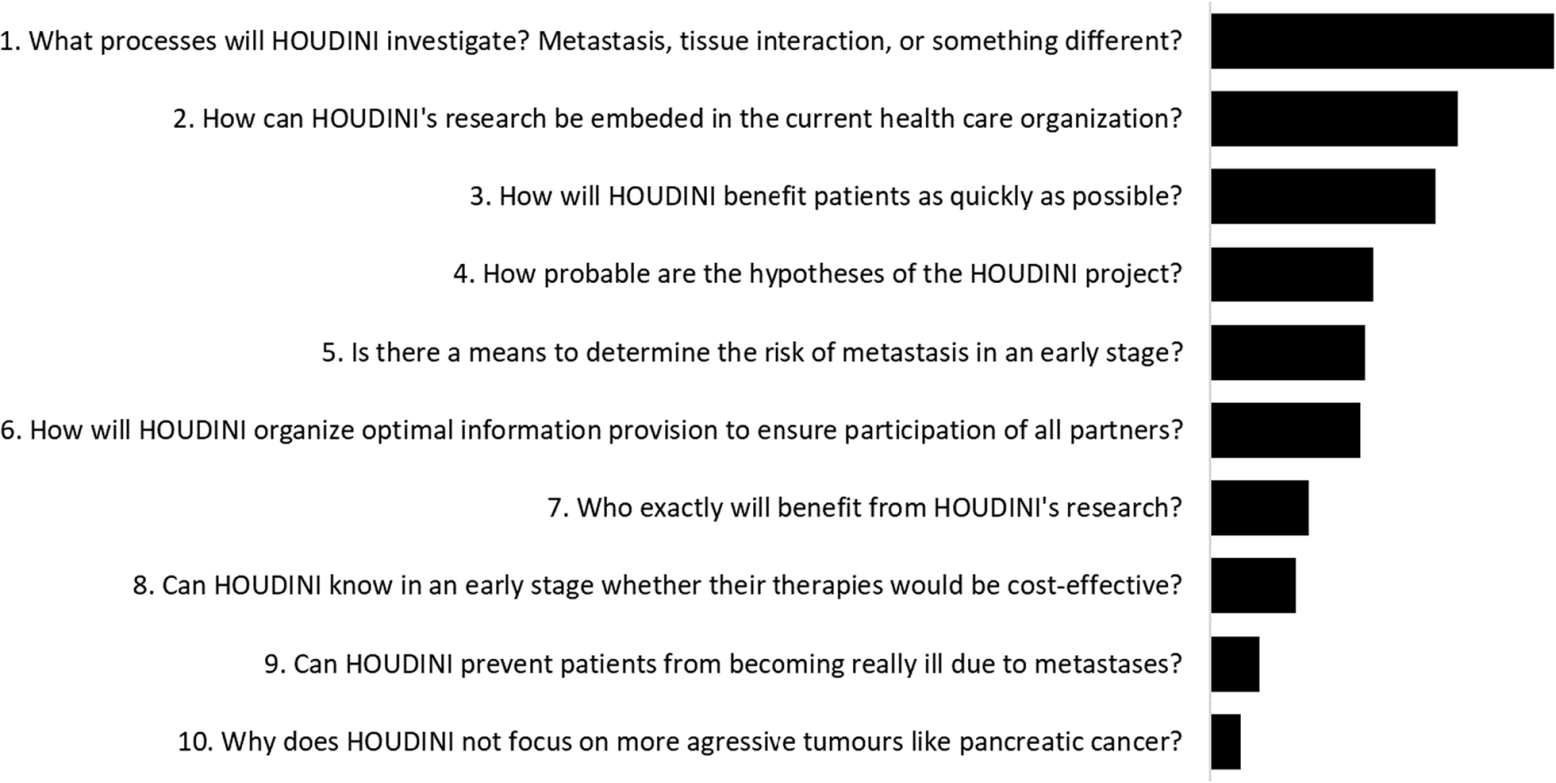
Top 10 questions for HOUDINI

The input from all four break out groups was collected and summarized in questions for the HOUDINI network. The participants of the workshop ranked these questions according to priority with a voting tool. The top 10 is shown in Fig. 3. The highest scoring question asked for better explanation of HOUDINI’s research objectives. Question 2 and 3 focus on the practical implications of HOUDINI’s research, how it will be organized such that the new technology can be used to help patients as soon as possible.

### Follow-up: basic science perspective

So far, we have argued that the apparent gap between the basic science and patient perspective on cancer can be bridged by a holistic PPI approach in metastasis research to foster mutual understanding and achieve increased treatment impact. We have reviewed the social science’s view on PPI and reported on a societal workshop as an example of PPI in practice. In accordance with the holistic scope of this paper, we subsequently discussed the results with basic and translational scientists to hear their view on metastatic cancer research and on the outcome of the PPI workshop.

In the course of the deliberations among HOUDINI’s research partners, it became clear that medical research, and particularly metastatic cancer research, is a sensitive field, dealing with an often lethal disease and harming the lives of many patients. While clinicians and translational researchers may be more driven by their wish to beat off cancer and cure patients, basic scientists are fascinated by cancer with the dedication to fully understand the disease in order to be able to tackle it. As one scientist phrased it: “Our fascination rather stems from admiration for nature itself. We look at cancer as a highly interesting and intriguing natural phenomenon. We see the beauty of cancer cells while at the same time realizing their destructive power. It is like with looking at a volcano eruption, it is awe-inspiring to see the power of nature, but at the same time we are afraid of its disruptive impact on its environment with lava streaming and destroying all other life.” Questions, such as why cancer develops and these aberrant uncontrolled cells survive are at the very heart of basic research, and constitute a strong motive for conducting their research aiming to tackle the disease. Like explained by a scientist, “when we look into the microscope, we are excited and wonder how cells make such beautiful structures and function in concordance to form tissues and organs. And we are intrigued to know why cancer cells are out of control, realizing these cells once killed a patient. But, in order to understand cancer, we need to look at these cells, nurture them and even look after them, so they can tell us what went wrong. With new developments to engage patients and their families in the management of their disease, we need to recognize and learn to appreciate these different perceptions not as opposing stand-points, but rather as two sides of the same medal, dedicated to beat cancer.”

The preliminary results of the workshop were also presented and discussed during a meeting of the network *in statu nascendi*, ten days after the societal workshop. This presentation resulted in a lively process of deliberation. One of the issues addressed was expectation management, which was elicited by the third question from Fig. 3: how can HOUDINI benefit patients as quickly as possible? This raises a dilemma. On the one hand, HOUDINI is devoted to basic research, aspiring to deepen our understanding of how and why metastatic cells affect healthy tissue, in order to establish new effective treatment options. This new knowledge is essential to define new targets for detection and therapy. While the time lines of generating such detailed knowledge and adoption of such results are distant, the ultimate aim of HOUDINI is dedicated to implementation of new detection methods and therapies. Here, adequate expectation management is essential to explain that careful research and testing of new treatment modalities prior to implementation is crucial, even though it may feel unnecessary slow. Importantly, overpromising may endanger the trustworthiness of the research project and of the science of metastasis more broadly. At the same time, research is a highly competitive arena and researchers often feel forced to exaggerate potential benefits in their proposals to secure funding. Closer interaction with patients via methods such as PPI could help in developing realistic and feasible research scenarios. Also, close collaboration with pharmaceutical industry and innovative companies is foreseen in the consortium to enable a rapid follow-up.

Finally, the question was addressed whether interactive forms of research such as PPI are feasible in practice. Involving societal stakeholders at the beginning and towards the end of the process is considered as beneficial to the project, but how to ensure longitudinal involvement of patients and practitioners? How to make PPI an intrinsic part of the methodology of science? Evidently, workshops such as the one analyzed in this paper, should not be one-time events, but should be incorporated in the research design to develop a long-term relationship of interaction and epistemic inclusion. Patients may be involved in research as donors of biomaterials, such as cells and tissues. In the context of projects such as HOUDINI, the aim is to develop reliable and representative models (based on organ-on-chip model systems), which may by-pass the need for animal models. Tissue donation offers patients the opportunity to become partners in the research, provided their donorship is not treated as a mere resource for research. It may open opportunities to participate in research in a more holistic manner, from designing the agenda to interpret the potential benefits of initial results.

## Discussion and conclusions

In this paper, we argued that a considerable gap exists between the lifeworld of cancer researchers and patients. To bridge this gap, PPI methodology is used across multiple clinical disciplines. PPI emerged against the backdrop of a broad movement which aims to include the practical and experiential knowledge of patients, physicians, caretakers, and others more intensively in research even at a relatively basic level. Especially in basic and translational research, it is difficult to implement PPI in a constructive way. Still, the metastasis research network HOUDINI took on this challenge in order to develop a holistic PPI approach, being interactive and inclusive. Therefore, a societal workshop was organized, which was the starting point of PPI implementation in the HOUDINI network. The workshop results were analysed and further enriched through interviews and participation in a network meeting. The resulting input was used to rethink and refine the HOUDINI approach, for instance by stressing the need for metastasis prediction tools, more effective therapies without severely impacting quality of life, and development of non-invasive tissue sampling methods. The workshop also created awareness about societal issues such as economic feasibility and unrealistic expectations, clear communication between stakeholders, and the anxiety brought about by metastasis. The participants, both basic scientists as well as patients, were engaged and respectful listening and understanding each other’s viewpoints. Crucial to the ultimate success of this PPI approach is to maintain and expand the contacts during later stages of HOUDINI’s research.

The number and background of the sixteen participants of the workshop was optimal for a mutual learning session fostering dialogue. Yet, the number was too small to be a cross section of society. The participants had diverse experience and expertise, but were mostly well-educated, white, western citizens. This composition of the group enabled a high-level discussion and delivered relevant input from engaged participants, but was not representative for the society in general. Limiting diversity and inclusiveness of PPI is a common and well-documented problem [46]. To be genuinely inclusive, HOUDINI should extend its scope and invite e.g. more patients and public from different cultural backgrounds or general practitioners with a clientele among the corresponding districts. We noticed that language and terminology already pose challenges within this well-educated group. It was almost as if multiple languages were spoken, each with a vocabulary of its own. If the scope of societal participation will be extended, even more effort should be put in understandable communication to clarify meanings and concepts during these meetings to involve all participants.

The overall experience of the workshop was that we unleashed a genuine dialogue in the sense that the arrow of communication was not one-sided, but interactive, and this also pertained to the knowledge dimension. Patients, physicians, societal partners shared their knowledge and experiences, and their questions and lessons were seen as relevant and informative for scientists. Vice versa, participants with practical knowledge actively questioned and commented on the scientific knowledge that was presented and discussed. Our conclusion is that PPI will foster the quality and potential relevance of research programs, but that it is a challenging goal to achieve maximal inclusion from all aspects of society. PPI also is challenging because it requires a change in current functioning and organization of research. Importantly, the key message of this work is that interactive research and epistemic inclusion is considered as valuable both by societal stakeholders and by researchers.

### Supplementary Information

**Additional file 1**: GRIPP2 short form.

## Data Availability

All data supporting the conclusions of this article are included in this article.

## Abbreviations

AIRR: Anticipation, inclusiveness, reflectiveness and responsiveness
ELSA: Ethical, legal and social aspects
Erasmus MC: Erasmus Medical Centre
EUR: Erasmus University Rotterdam
HOUDINI: Holistic approach to UnderstanD and target the metastatic Niche
JLA: James Lind Alliance
PPI: Patient and public involvement
PSIDER: Pluripotent Stem cells for Inherited Diseases and Embryonic Research
PSP: Priority setting partnership
RRI: Responsible research innovation
TU Delft: Technical University Delft
