# Supplementary material for: Awe and anxiety for cancer cells: connecting scientists and patients in a holistic approach of metastasis research
Source: Res Involv Engagem. 2023 Sep 26;9:85. doi: 10.1186/s40900-023-00498-3 (PMC10523712; doi:10.1186/s40900-023-00498-3)
Supplement: Supplementary file 1 — Additional file 1: GRIPP2 short form. [file 40900_2023_498_MOESM1_ESM.docx]

GRIPP2 short form

| **Section and Topic** | **Item** | **Reported on page No** |
| --- | --- | --- |
| 1: Aim | Report the aim of PPI in the study | 6-7 |
| 2: Methods | Provide a clear description of the methods used for PPI in the study | 14-15 |
| 3: Study results | Outcomes—Report the results of PPI in the study, including both positive and negative outcomes | 15-21 |
| 4: Discussion and conclusions | Outcomes—Comment on the extent to which PPI influenced the study overall. Describe positive and negative effects | 21-25 |
| 5:  Reflections/critical perspective | Comment critically on the study, reflecting on the things that went well and those that did not, so others can learn from this experience | 23-25 |

PPI=patient and public involvement
